# Symptomatic Hypocalcemia due to Nutritional Vitamin D Deficiency in Three Adolescents during the COVID-19 Pandemic

**DOI:** 10.1155/2023/3588196

**Published:** 2023-10-19

**Authors:** Sabitha Sasidharan Pillai, Lisa Swartz Topor

**Affiliations:** ^1^Division of Pediatric Endocrinology, Hasbro Children's Hospital, Providence, USA; ^2^Department of Pediatrics, The Warren Alpert Medical School of Brown University, Providence, USA

## Abstract

**Background:**

Symptomatic hypocalcemia secondary to vitamin D deficiency (VDD) is rare among adolescents without underlying medical disorders, but its prevalence is higher in known risk populations. We report on three adolescent males with low nutritional intake of vitamin D and calcium and limited sun exposure who presented with hypocalcemic tetany and muscle cramps due to VDD during the COVID-19 pandemic. *Case Reports*. Three adolescent males (age range 14 to 16 years) presented with symptomatic hypocalcemia: paresthesia, carpopedal spasms, and muscle cramps. All reported limited dairy intake and sun exposure. Laboratory studies showed mean ionized calcium (iCa) 2.73 mg/dl (range 2.69–2.8), mean phosphorus 4.17 mg/dl (range, 3–5.4), mean parathyroid hormone (PTH) 431.67 pg/mL (range, 320–527), and mean 25-hydroxyvitamin D (25(OH)D) 7.37 ng/mL (range 5.3–10.8). All the patients presented during the COVID-19 pandemic, and one had COVID-19 infection. All were treated with oral calcium and high dose ergocalciferol. Patients 2 and 3 were also treated with intravenous calcium gluconate infusion and oral calcitriol.

**Conclusion:**

Severe VDD with symptomatic hypocalcemia can occur among adolescents without underlying medical diagnoses due to dietary and behavioral habits that limit nutritional intake and sun exposure. Risk factors of the patients may have been potentiated by pandemic-related behaviors such as more time indoors at home related to social distancing, as well as diets with limited nutrient intake. Adolescents presenting with nonspecific musculoskeletal symptoms should be screened for VDD and hypocalcemia. Appropriate treatment and preventive measures can stop immediate and long-term complications.

## 1. Introduction

Vitamin D deficiency (VDD) is one of the most common nutritional deficiencies worldwide [1]. Various epidemiological studies report that prevalence of vitamin D deficiency (serum 25-hydroxyvitamin D (25 (OH)D) level of <20 ng/ml [2]) among healthy adolescents in the USA ranges from 21% to 42% based on geographical location, sun exposure, ethnicity, gender, body mass index (BMI), and dietary factors [3]. Hypocalcemia and low 25(OH)D levels have also been reported among patients with COVID-19 infection [4]. While younger children with VDD may present with rachitic changes or hypocalcemia symptoms such as seizures, twitching, cramping, or rarely laryngospasm, adolescents with VDD may be asymptomatic or may have nonspecific symptoms such as joint pains, myalgias, muscle weakness, or muscle cramps [5]. Facial twitches and carpopedal spasms are rare manifestations of VDD among adolescents [5]. We describe three adolescent males without prior medical diagnoses who presented with hypocalcemic tetany due to VDD and limited dietary Ca intake [6].

## 2. Case Reports (Table 1)

  Patient 1: A 16 years 5-month-old non-Hispanic Black male presented with tingling sensations, initially involving hands and feet and later spreading to his entire body over the week prior to presentation and generalized muscle cramps. Polymerase chain reaction (PCR) testing for COVID-19 infection was negative at the time of presentation. His past medical history was significant for evaluation of malnutrition by gastroenterology with work up including unremarkable colonoscopy and normal inflammatory markers. His diet included bread, popcorn, pizzas, barbeque chicken, and chicken nuggets and lacked dairy products. He had an underweight BMI at the 1.1 percentile (target BMI ≥5^th^ to < 85^th^ percentile [7]). He had stable vitals and positive Chvostek sign [6].
  Patient 2: A 14 years 2.5-month-old Hispanic male presented with worsening muscle cramps of 3 months duration. Symptoms started while recovering from COVID-19 infection diagnosed by PCR testing, initially as leg cramps lasting for few seconds which worsened in terms of duration, frequency, and severity over the next 3 months to multiple daily episodes of cramps involving his jaw, arms, and legs, and carpal spasms lasting about an hour. He also endorsed tingling sensations all over the body. He was an otherwise healthy child. His diet included bread, fruits, and vegetables mainly with occasional cheese, yogurt, and chicken nuggets. Examination revealed a very anxious adolescent with tachycardia, elevated blood pressure (122/78 mm Hg), BMI at the 86.5 percentile (overweight), and positive Trousseau sign. He was negative for COVID-19 infection at presentation [6].
  Patient 3: A 16 years 3-month-old Hispanic male presented with abdominal pain, vomiting, paresthesia, and tingling sensations involving the upper extremities of 2 days duration. His past medical history was noncontributory. His diet included pizza and chicken nuggets and lacked dairy products. On examination, he was febrile, normotensive with bilateral carpal spasms. His BMI was at the 55.1 percentile. His PCR test for COVID-19 was positive [6].

All reported limited sun exposure owing to the COVID-19 pandemic and more time spent indoors. None of them were taking calcium or vitamin D supplements.

Laboratory work up confirmed VDD in all patients. Patient 2 also had iron deficiency anemia. Family members were not screened for VDD or hypocalcemia. Patient 1 was started on oral calcium carbonate (elemental calcium 100 mg/kg/day); vitamin D3 5000 units weekly for eight weeks was initiated on day 2, once serum calcium was above 7 mg/dl. He was discharged on day 2. Patients 2 and 3 were started on continuous calcium gluconate infusion (2 gram of calcium/L) along with oral calcium carbonate (100 mg of elemental Ca/kg/day), oral calcitriol 0.25 mcg twice daily for 3–6 days, and ergocalciferol 50,000 units once weekly for 8 weeks. For patients 2 and 3, calcium gluconate infusion was weaned and discontinued after two days once serum calcium concentrations improved. Following hospital discharge, patients 2 and 3 remained asymptomatic with normal Ca and Vitamin D levels. Patient 1 was lost to follow up [6].

## 3. Discussion

We describe three adolescent males who resided at a northern latitude and presented with symptomatic hypocalcemia due to VDD and low dietary Ca intake during late winter and early spring months. Limited sun exposure, dietary habits, northern geographic latitude, winter season, and pigmented skin were contributing factors in the development of VDD. Infection may also have played a role, as two patients developed hypocalcemia symptoms in the setting of COVID-19 infection: one presented with active COVID-19 infection and one developed progressive hypocalcemia symptoms that began 3 months prior in the setting of COVID-19 infection.

The main source of Vitamin D is D3 synthesized within the human body via the action of ultraviolet B radiation on 7-dehydrocholesterol in skin. Thus, the vitamin D3 level is influenced by environmental (latitude and season) and individual (melanin in skin, sun exposure, use of sunscreen, clothing that covers the entire body, and obesity) factors. The rest is derived from dietary sources such as oily fish, eggs, and fortified food such as milk and cereals [8, 9].

Increasing the zenith angle of sun at northern latitudes during wintertime precludes cutaneous synthesis of Vitamin D3 due to less UVB radiation reaching the skin, putting adolescents in northeast area of the US at a greater risk for VDD [8, 10]. During these periods of inadequate sun exposure, dietary factors become crucial in maintaining optimal vitamin D level [8]. A study on the prevalence of VDD among 307 healthy adolescents from Boston, USA, reported higher prevalence of hypovitaminosis D during winter and spring compared to summer and fall [10]. One of our patients presented during late winter and the remaining 2 during early spring. Recently, there has been a reduction in the milk intake by adolescents, partly due to increased consumption of sugary beverages [8, 10]. Removal of this alternate source for Vitamin D further increases the risk of VDD. All three patients in our study had restricted dietary intake of vitamin D and calcium: two favored fast foods and one ate a mostly vegan diet.

Symptomatic VDD is rare among adolescents [7] though it has been described in adolescents with risk factors such as restricted diets or developmental disorders [11] or limited sun exposure for cultural and religious reasons [9]. A recent report from New York described two healthy young adolescents who presented with seizures and right knee pain, respectively, due to severe VDD which was attributed to limited diet and reduced sun exposure during COVID-19 pandemic [12]. Our patients were older than those reported by the New York study, and all presented with tetany. Two patients in the current report had a positive Trousseau sign (carpopedal spasm occurring after a few minutes of inflation of a sphygmomanometer cuff above systolic blood pressure) and one had positive Chvostek sign (twitching of facial muscles produced by tapping over the facial nerve). Risk of VDD and increased BMI have a linear relationship, due to lack of bioavailable vitamin D secondary to increased fat deposition of Vitamin D [13], which may have been a risk factor for one of our patients with BMI in overweight range.

Vitamin D sufficiency during adolescence is important as significant bone mineral accrual occurs during adolescence, with up to 40% of adult bone mineralization occurring during peak bone growth velocity. Inadequate bone mineralization due to VDD during this crucial period might adversely affect bone mineralization, thereby increasing the risk of osteoporosis later in life [14].

Hypocalcemia stimulates parathyroid hormone secretion with the goal of restoring normal calcium levels. Secondary hyperparathyroidism enhances bone resorption, which leads to maintenance of serum calcium levels initially, but with the consequences of bone demineralization and phosphaturia. In the absence of restoration of normal serum calcium or vitamin D levels via increased intake or supplementation, the hyperparathyroidism eventually leads to depletion of bone calcium stores. Phosphaturia with resultant hypophosphatemia causes poorly mineralized osteoid formation (osteomalacia). In those with open growth plates, rickets also develops due to defective mineralization of cartilage at the epiphyses [15]. The laboratory findings in our patients, including VDD, hypocalcemia, elevated PTH, and elevated alkaline phosphatase, are indicative of hypomineralization of bone.

Patient 1 had an elevated phosphorus level. Though VDD is usually associated with low phosphorus level, rarely hyperphosphatemia can occur due to acquired pseudohypoparathyroidism from hypocalcemia of VDD and/or desensitization of receptors from chronically increased parathyroid hormone values [16].

In order to prevent VDD and its sequalae, daily supplementation with vitamin D 400 international units and calcium are recommended for healthy adolescents whose diet does not include vitamin D and calcium, especially those with limited sun exposure [17].

## 4. Conclusion

Severe VDD with symptomatic hypocalcemia may occur in adolescents without prior medical diagnoses due to lack of dietary intake combined with lack of sun exposure. Adolescents presenting with nonspecific musculoskeletal symptoms should be screened for VDD and hypocalcemia. Appropriate treatment and preventive measures can stop immediate and long-term complications.

## Figures and Tables

**Table 1:** Clinical and laboratory parameters of the patients.

Characteristics and laboratory values	Patient 1	Patient 2	Patient 3
Age, gender	16 y 5 mo, male	14 y 2 mo, male	16 y 3 mo, male
Presenting symptoms	Muscle cramps and paresthesia, COVID-19 PCR negative	Worsening muscle cramps, paresthesia, and carpal spasms	Fever, abdominal pain, nausea, and vomiting, paresthesia, carpal spasms, and COVID-19 PCR positive
Signs at admission	Chvostek's sign	Trousseau's sign	Trousseau's sign
BMI %ile (Z score)	1.13 (−2.28)	86.54 (1.10)	55.10 (0.13)
Predisposing factors	Limited diet with no dairy products. Decreased sun exposure. Pigmented skin	Limited diet with no dairy products, mostly vegan. Decreased sun exposure. Pigmented skin	Limited diet with no dairy products. Decreased sun exposure. Pigmented skin
Serum calcium (8.5–10.5 mg/dl)	6.5	6.6	5.2
Albumin (3.1–4.8 g/dl)	4.2	4.4	4.5
Ionized calcium (4.2–5.2 mg/dL)	2.80	2.70	2.69
Serum magnesium (1.3–1.9 mEq/L)	1.5	1.7	1.3
Serum phosphorus (2.4–4.8 mg/dl)	5.4	4.1	3
PTH (18–80 pg/mL)	527	448	320
25 (OH) D (30–100 ng/mL)	10.8	5.3	6
1,25 (OH)_2_ vitamin D (19.9–79.3 pg/mL)	—	35.4	—
EKG	Normal sinus rhythm	Normal sinus rhythm	Normal sinus rhythm. Prolonged QTc with delayed onset of T wave
Alkaline phosphatase (13 to <15 years-127–517 IU/L, 15 to <17 years-89–365 IU/L)	637	558	421
Creatine kinase (20–210 IU/L)	392	—	443

PTH: parathyroid hormone, EKG: electrocardiogram.

## Data Availability

All the data used to support the findings of this study are included within the article.

## Abbreviations

BMI: Body mass index
Ca: Calcium
iCa: Ionized calcium
CK: Creatine kinase
Lab: Laboratory
PTH: Parathyroid hormone
P: Phosphorus
Mg: Magnesium
25(OH)D: 25-Hydroxyvitamin D
UVB: Ultraviolet B
VDD: Vitamin D deficiency
PCR: Polymerase chain reaction.
